# Characterization of *PsmiR319* during flower development in early- and late-flowering tree peonies cultivars

**DOI:** 10.1080/15592324.2022.2120303

**Published:** 2022-10-06

**Authors:** Chenjie Zhang, Jiajia Shen, Can Wang, Zhanying Wang, Lili Guo, Xiaogai Hou

**Affiliations:** a College of Agriculture/Tree Peony, Henan University of Science and Technology, Luoyang China; b Peony Research Institute, Luoyang Academy of Agricultural and Forestry Sciences, Luoyang China

**Keywords:** Tree peony, *PsmiR319*, flower development, early and late flowering, expression analysis

## Abstract

The flowering period is the most important ornamental trait of tree peony, while industrial development of tree peony has been limited by short flowering period. miR319 plays an important regulatory role in plant flowering. In the current study, the expression characteristics and evolution of *PsmiR319* in tree peony flowering was explored using ‘Feng Dan’ and ‘Lian He’, which are early-flowering and late-flowering varieties of tree peony, respectively. The structure, evolution, and target(s) of *PsmiR319* were analyzed by bioinformatics. Evolution analysis showed that pre-*PsmiR319* was distributed in 41 plant species, among which the length of the precursor sequence exhibited marked differences (between 52 and 308 bp). Pre-*PsmiR319* of tree peony was located close to the corresponding sequences of *Linum usitatissimum* and *Picea abies* in the phylogenetic tree, and in addition, could form a typical hairpin structure including a mature body with a length of 20 bp located on the 3p arm and part of the loop sequence. The mature sequence of miR319 was highly conserved among different species. Target genes of *PsmiR319* include MYB-related transcription factor in tree peony. Expression of *PsmiR319*, assayed by qRT-PCR, differed between ‘Feng Dan’ and ‘Lian He’ during different flower development periods. *PsmiR319* and its target gene showed a negative expression regulation relationship during the periods of CE (color exposure), BS (blooming stage), IF (initial flowering), and HO (half opening) in the early-flowering ‘Feng Dan’, and the same in FB (Full blooming) periods of late-flowering ‘Lian He’. Findings from this study provide a reference for further investigation into the mechanism of miR319 in the development of different varieties of tree peony.

## 1 Introduction

Tree peony (*Paeonia suffruticosa* Andrews.), belonging to section *Moutan*, genus *Paeonia*, and family Paeoniaceae, is a woody plant^1^ with important ornamental, medicinal, and edible characteristics, and is therefore an economically valuable, multi-purpose plant.^2,3^ Tree peony is a very popular traditional ornamental plant in China and is also appreciated internationally due to its large showy flowers.^4^ However, development of the tree peony industry has been restrained by the short flowering period of this plant. Different flowering times and long flowering periods are crucial for enhancing the applications and production of tree peony. Thus, understanding of the molecular mechanism of flowering time in tree peony may provide a theoretical basis for flowering regulation and breeding of the plant.

Selecting the right time to bloom is crucial for angiosperm plants to complete sexual reproduction.^5^ Most annual or biennial plants flower only once in their life cycle, whereas woody perennials undergo repeated cycles of vegetative and reproductive growth.^6^ Regulation of flowering time plays an important role in perennial woody plants, which determines the reproductive and genetic ability of plants, and is also valuable for long-term fruit or flower production.^7^ A complex genetic regulatory network is utilized by plants to perceive and integrate changes in external environmental factors and endogenous factors into signaling pathways that maintain strict control of flowering.^8^ Plants have formed complex signal transduction pathways through long-term biological evolution, and microRNAs (miRNAs) play a crucial role by controlling the expression of key flowering genes in this huge regulatory network.^9–11^

miRNA is a type of endogenous non-coding RNA with regulatory functions that is found in eukaryotes and consists of 20–26 nucleotides.^12^ Many studies have shown that mature miRNA, derived from stem-loop precursor sequences (pre-miRNAs), regulates gene expression at the post-transcriptional processing stage.^13^ miRNA functions in plant growth and development, cell differentiation, apoptosis, lipid metabolism, hormone secretion, signal transduction, and the stress response by truncating the target gene or inhibiting translation of the target gene.^14–16^ In addition, miRNAs are relatively conserved in species evolution, which is indicative of the importance of their functions. miRNAs were shown to play vital roles in the flowering process of plants almost two decades ago.^17^ Subsequently, several miRNAs that regulate plant flowering time have been reported. In *Arabidopsis thaliana*, mir156-regulated *SPL* transcription factors define an endogenous flowering pathway,^18^ miRNA172 mediates photoperiodic flowering independent of *CONSTANS*,^20^ and miR393 affects developmental timing and patterning by *TAS3* ta-siRNA.^21^

miR319, one of the more thoroughly researched miRNA representatives related to plant development, is a conserved plant miRNA and plays a regulatory role in various biological pathways of plant growth and development,^22^ including seed sprouting,^23^ leaf development,^24,25,^ flowering,^26,27^ flower-organ growth,^28–30^ hormone signaling,^31^ and response to environmental stresses such as cold, salt, and drought.^10,19,32–34^ However, information on miR319 of tree peony is limited, and how this molecule regulates the flowering process of peony has yet to be elucidated.

Based on the preliminary data of our laboratory (unpublished data), we have established miRNA databases for different varieties and different flower development stages of tree peony. The present investigation was designed to analyze the structure and evolution of miR319 using bioinformatics, and furthermore identify the expression of *PsmiRNA* and its target genes in different flower development stages of different species of tree peony. Findings from the study lay a theoretical foundation for *PsmiR319* to regulate the flowering period of tree peony.

## 2 Materials and methods

### 2.1 Plant material and growth conditions

**Figure 1.**
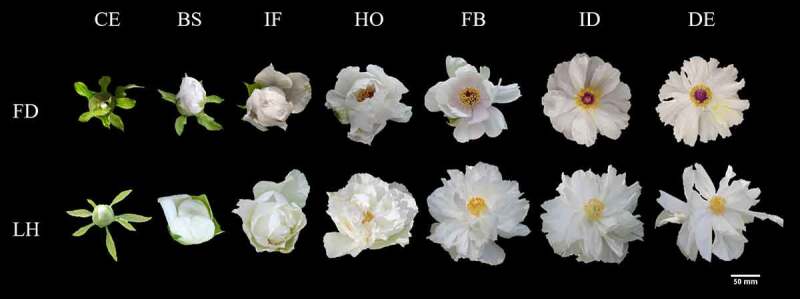
Form characteristics during different flower development stages of two varieties of tree peony.

Two tree peony species differing in flowering time were used in this study. The early-flowering specie ‘Feng Dan’ (*Paeonia ostii* T. Hong et J. X. Zhang var. lishizhenii B. A. Shen) and late-flowering specie ‘Lian He’ (*Paeonia suffruticosa* Andr. cv. Lian He) were collected from Sui and Tang Dynasties City Ruins Botanical Garden, Luoyang, Henan Province, China (112°45′36″ E, 112°45′36″ N). The region had a medium-latitude climate. Under field conditions, petal samples at seven different development periods (CE: color exposure; BS: blooming stage; IF: initial flowering; HO: half opening; FB: full blooming; ID: initial decay; DE: decay) of eight-year-old plants with consistent growth after planting in the same ecological environment were collected and three replicates were taken for each period. Only the petals located on the top of branches were collected. The samples were immediately frozen in liquid nitrogen and stored at −80°C until RNA and miRNA extraction. The form of the seven different flower development stages of the two tree peony varieties is depicted in Figure 1.

### 2.2 RNA isolation and complementary DNA (cDNA) synthesis

Total RNA was extracted from collected petals using a RNAprep Pure Plant Kit (Polysaccharides&Polyphenolics-rich, TIANGEN). RNA quality and purity were checked by 1% agarose gel electrophoresis, using the DL2000 DNA Marker (TaKaRa) as a size indicator. RNA concentrations and ratios of absorbance at 260 nm to that at 280 nm (260/280) were determined using a NanoDrop 1000 spectrophotometer (Implen, Germany). Total RNA was reverse transcribed into cDNA according to the steps of PrimeScript RT reagent Kit with gDNA Eraser (TaKaRa). PCR amplification was conducted in 20 µL reaction mixtures containing 10 µL total RNA of erasing gDNA, 1 μL PrimeScript RT Enzyme Mix 1, 1 µL RT Primer Mix, 4 μL 5× PrimeScript Buffer 2 (for Real Time), and ddH_2_O to adjust the volume. Cycling conditions were 37°C for 15 min, then 85°C for 5 s.

### 2.3 miRNA isolation and cDNA synthesis

Total miRNA was extracted using a miRcute Plant miRNA Isolation Kit (TIANGEN). miRNA quality and purity were checked using the same method as for total RNA. miRNA cDNA, which was later used as a template for expression studies, was synthesized using a miRcute Plus miRNA First-Strand cDNA Kit (TIANGEN). The reaction system comprised 4 μL total miRNA, 10 μL 2× miRNA RT Reaction Buffer, 2 μL miRNA RT Enzyme Mix, and ddH2O to adjust the volume to 20 μL. A reverse transcription program of 42°C for 15 min and 95°C for 3 min was utilized.

### 2.4 Species distribution and phylogenetic tree reconstruction of plant miR319

Pre-miR319 and mature miR319 sequences of all plants registered on the miRBase database (https://mirbase.org/) were used for bioinformatics analysis. The species with pre-miR319 that had been registered were classified and counted. MEGA6.0 software was used to reconstruct a phylogenetic tree of miR319 precursor sequences via the neighbor-joining method.

### 2.5 Mature miR319 sequence analysis and multiple alignment

Mature sequences of all members of the miR319 gene family from the miRBase database (https://mirbase.org/) were downloaded and analyzed. DNAMAN was used to compare and analyze multiple sequences.

### 2.6 Prediction of the secondary structure of pre-miRNAs and conservation analysis

Pre*-PsmiR319* of tree peony was used for prediction of the secondary structure using RNAfold software (http://rna.tbi.univie.ac.at/cgi-bin/RNAWebSuite/RNAfold.cgi) and the characteristics of the mature body position were analyzed. The conserved nucleotide sequence of pre*-PsmiR319* was analyzed online using Rfam12.0 (http://rfam.xfam.org).

### 2.7 Target gene prediction of miR319

miR319 target genes of three model plants-*A. thaliana, Oryza sativa*, and *Nicotiana tabacum* – and *Paeonia suffruticosa* were predicted using online target site analysis software psRNATarget (http://plantgrn.noble.org/psRNATarget/) combined with BLAST (https://blast.ncbi. nlm.nih.gov/Blast.cgi). *PsmiR319*-regulated mRNA target sites were found by degradome sequencing of ‘Feng Dan’ and ‘Lian He’ and specific nucleotide sequence of psu.T.00032704 that cleaved by *PsmiR319* was predicted using online target site analysis software psRNATarget (http://plantgrn.noble.org/psRNATarget/).

### 2.8 Validation using quantitative real-time PCR (qRT-PCR)

The expression of miRNA and corresponding target genes of different varieties of tree peony was analyzed using qRT-PCR. Primers (Table 1) were designed using Primer Premier 5 and the Oligo 7 Analyzer Tool. The reverse primers of miRNA were provided by the reagent kit (TIANGEN). Based on the screening results of internal reference genes in development of tree peony flowers (unpublished), EF1-α gene was used as an internal control to normalize gene expression. U6 was used as an internal control to normalize miRNA expression.^35^ A TB Green Premix Ex Taq II kit (TaKaRa) was used for qRT-PCR, while a miRcute Plus miRNA qPCR Detection Kit (SYBR Green) (TIANGEN) was used for analysis of miRNA content. Reactions were run on the CFX96 Real-Time PCR machine (Bio-Rad, American) with three technical replicates. Results were calculated using the formula of Relative Expression = 2^−ΔΔCt^. Each expression profile is shown as the log2 value of the fold-change. Significant differences within groups were analyzed by SPSS, i.e. miRNA and target genes are compared individually.

**Table 1.** Primers used in qRT-PCR assays.

primer	Primer sequence(5’-3’)	Annealing temperature(θ/°C)	Annealing time(t/s)	qPCR assay purpose
miR319-For	GTCCTGCTGCCATCTCATGCAT	68	34	miR319
U6-For	ACAGAGAAGATTAGCATGGCC	68	34	miR319
PsMYB-For	TAGTGGGTCAGTCTCTTCGGG	66	30	MYB
PsMYB-Rev	GATAAGGTTTCGGGGTGTTGT	64	30	MYB
EF1-α-For	CCGCCAGAGAGGCTGCTAAT	64	30	reference gene
EF1-α-Rev	GCAATGTGGGAAGTGTGGCA	62	30	reference gene

## 3 Results

### 3.1 Plant miR319 species distribution and phylogenetic tree reconstruction

Pre-miR319 of 41 plant species were logged on the miRBase database (version 22.1), and statistics of botanical classification were derived for these 41 species and tree peony (Table S1). miR319 precursors were distributed in four divisions of Angiosperms, Gymnospermae, Pteridophyta, and Bryophyta, including six classes, 20 orders, 22 families, and 40 genera. Multiple (≥2) miR319 family members were present in *Medicago truncatula, Vigna unguiculata, Malus domestica, Prunus amygdalus, Arabidopsis lyrata, A. thaliana, Cucumis melo, Linum usitatissimum, Manihot esculenta, Ricinus communis, N. tabacum, Lycopersicon esculentum, Solanum tuberosum, Populus trichocarpa, Vitis vinifera, Amborella trichopoda, Sorghum bicolor, Brachypodium distachyon, O. sativa, Zea mays, Asparagus officinalis, Camelina sativa, Picea abies*, and *Physcomitrella patens*. The pre-miR319 sequence was markedly different among different species; the shortest sequence (52 bp) was discovered in *P. abies*, while the longest was discovered in *M. pumila* (308 bp).

**Figure 2.**
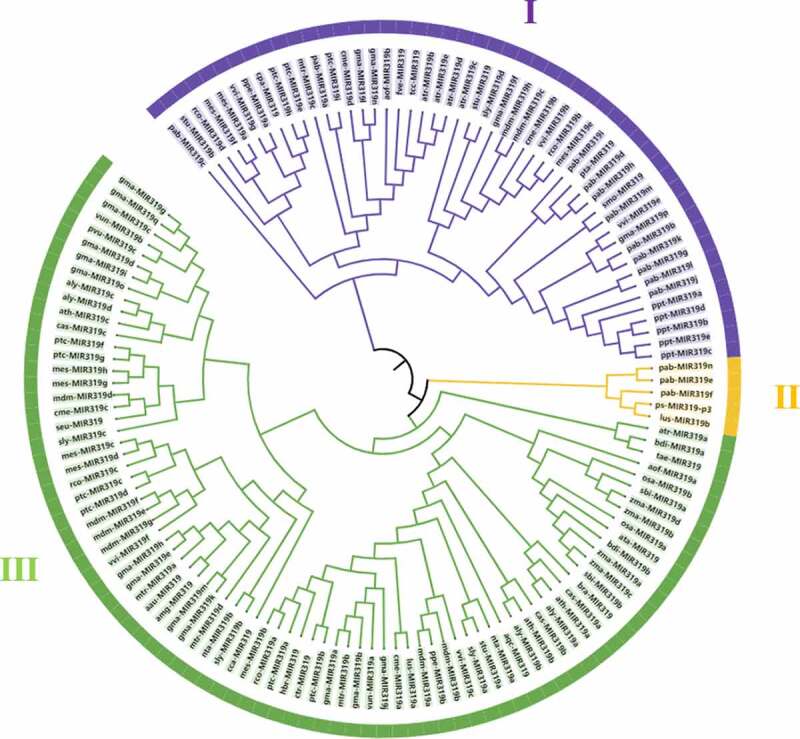
Phylogenetic tree of pre-miR319 sequences from different plant species.

To further comprehend the evolutionary characteristics of plant miR319, MEGA6.0 was used to reconstruct a phylogenetic tree of the precursor sequences (Figure 2). The species were roughly divided into three categories – pre-*PsmiR319* of tree peony and partial precursor sequences of *L. usitatissimum* (MIR319b) and *P. abies* (MIR319e/f/n) were clustered into a branch, indicating tree peony is more closely related to these species. The two plants are *Linaceae* and *Pinaceae*, which indicates the evolutionary conservation and rich diversity of miR319. Sequences of *P. patens* (MIR319a/b/c/d/e) in Bryophyta, *Selaginella moellendorffii* (MIR319) in Pteridophyta, *P. abies* (MIR319a/b/c/d/g/h/i/j/k/l/m) and *Pinus taeda* (MIR319) in Gymnospermae, *Glycine max* (MIR319f/l/n/p), *M. truncatula* (MIR319c), *M. domestica* (MIR319c/h), *Prunus persica* (MIR319a), *Fragaria vesca* (MIR319), *C. melo* (MIR319b/d), *Carica papaya* (MIR319), *M. esculenta* (MIR319a/e/f), *R. communis* (MIR319b/d), *Solanum Lycopersicum* (MIR319d), *S. tuberosum* (MIR319/b), *P. trichocarpa* (MIR319e/h/i), *Theobroma cacao* (MIR319), *V. vinifera* (MIR319b/e/g), *A. trichopoda* (MIR319b/c/d/e) and *A. officinalis* (MIR319b) were clustered into another branch; and then the pre-miR319 sequences of *A. trichopoda* (MIR319a), *B. distachyon* (MIR319a/b), *Triticum aestivum* (MIR319), *A. officinalis* (MIR319a), *O. sativa* (MIR319a/b), *S. bicolor* (MIR319a/b), Z. mays (MIR319a/b/c/d), *Aegilops tauschii* (MIR319), *Brassica rapa* (MIR319), *C. sativa* (MIR319a/b), *A. thaliana* (MIR319a/b/c), *A. lyrata* (MIR319a/b/c/d), *Aquilegia caerulea* (MIR319), *N. tabacum* (MIR319a/b), *S. tuberosum* (MIR319a), *S. Lycopersicum* (MIR319a/b/c), *V. vinifera* (MIR319c/f), *M. domestica* (MIR319a/b/d/e/f/g), *P. persica* (MIR319b), *L. usitatissimum* (MIR319a), *C. melo* (MIR319a/c), *G. max* (MIR319a/b/c/d/e/g/h/i/j/k/m/o/q/), *V. unguiculata* (MIR319a), *M. truncatula* (MIR319a/b/d), *P. trichocarpa* (MIR319a/b/c/d/f/g), *Citrus trifoliata* (MIR319), *Hevea brasiliensis* (MIR319), *R. communis* (MIR319a/c), *M. esculenta* (MIR319b/c/d/g/h), *Cynara cardunculus* (MIR319), *Acacia mangium* (MIR319), *Acacia auriculiformis* (MIR319), *Salicornia europaea* (MIR319), *C. sativa* (MIR319c), *Phaseolus vulgaris* (MIR319c) and *V. unguiculata* (MIR319b) were gathered together. The precursor sequence of *PsmiR319* of tree peony was located far from those of other angiosperms in the phylogenetic tree, indicating that there were differences between species, and that the origin, which is affected by many factors, was relatively unique.

### 3.2 Mature miR319 sequence analysis and multiple alignment

The sequences of mature miR319 from plants were analyzed to further explore the sequence characteristics of this miRNA. Mature miR319 sequences of all species retrieved in the miRBase database were combined with the *PsmiR319* sequence of tree peony and analyzed (Table S2). The copy number of mature miR319 is different in different plants. *G. max* had the highest number of miR319 (17), while *P. abies* was second with 14, and *M. pumila* contained 10. Eight mature miRNAs were detected for *Arabidopsis lyrate, M. truncatula, M. esculenta, P. trichocarpa*, and *Z. mays*; seven for *P. patens*; five for *L. esculentum, S. tuberosum*, and *A. trichopoda*; four for *C. melo, R. communis*, and *O. sativa*; three for *A. thaliana, C. sativa*, and *B. distachyon*; two for *B. rapa, L. usitatissimum, N. tabacum, P. amygdalus, V. unguiculata, S. bicolor, A. officinalis*, and *A. tauschii Coss*; and the remaining plants all contained one miR319. Mature sequences from the 5p and 3p arms were found in *A. tauschii, A. lyrata, B. rapa, B. distachyon, M. truncatula, M. domestica, O. sativa, P. patens, S. tuberosum, S. Lycopersicum*, and *Z. mays*.

**Figure 3.**
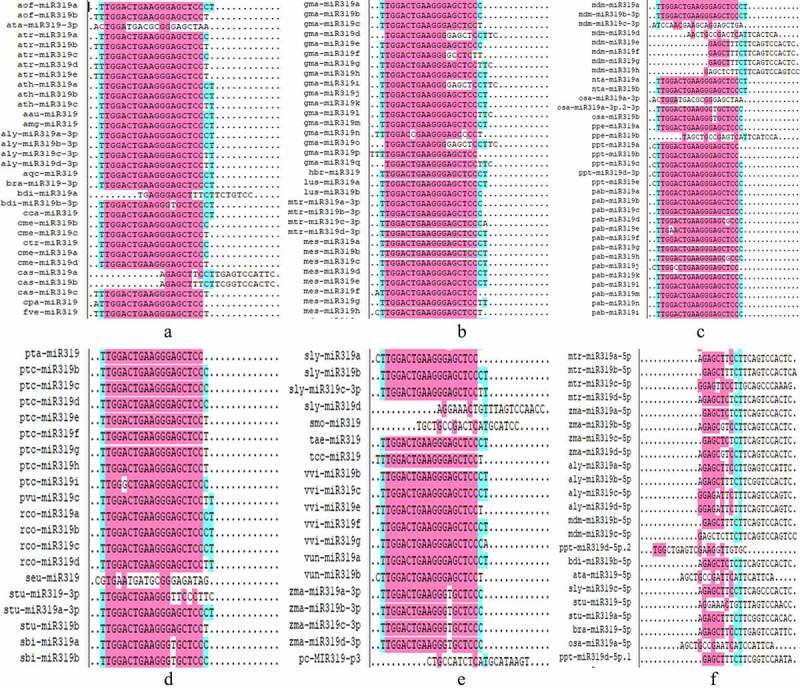
Multi-sequence comparison results of mature miR319 in plants.

All mature miR319 sequences were subjected to multiple sequence comparison analysis (Figure 3), and this showed that the mature sequences from the 5p arm were markedly different. The mature miR319 sequences from the 3p arm and were relatively conservative; the same miR319 sequence (UTGGACTGAAGGGAGCTCCCT) was found in the species *A. auriculiformis, A. mangium, A. lyrata, Aquilegia viridiflora, B. rapa, B. distachyon, C. cardunculus, L. usitatissimum, G. max, H. brasiliensis, S. Lycopersicum, M. esculenta, R. communis, S. tuberosum, M. domestica, T. aestivum, N. tabacum, V. vinifera*, and *V. unguiculata*.

### 3.3 *Prediction of the secondary structure of pre-*PsmiR319 *of tree peony*

**Figure 4.**
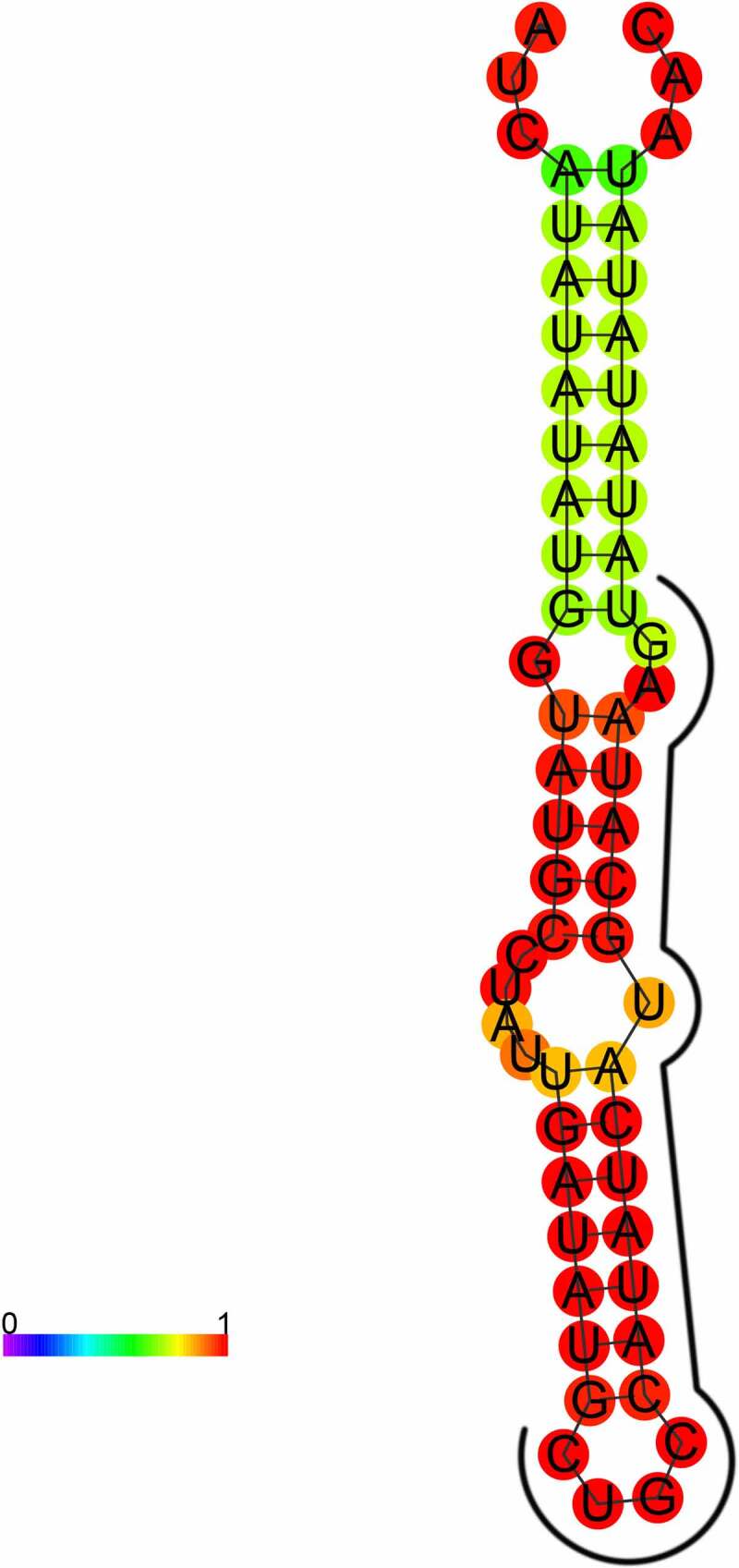
Mature and precursor sequence and the predicted stem-loop structures of *PsmiR319* in tree peony.

The secondary structure prediction revealed that the precursor of *PsmiR319* could form a typical hairpin structure, with the mature sequence including part of the 3p arm sequence and part of the loop sequence (Figure 4). Conservation of the secondary structure of pre-*PsmiR319* was analyzed by Rfam12.0 online and no sequence was detected.

### 3.4 Target gene prediction of miR319

**Figure 5.**
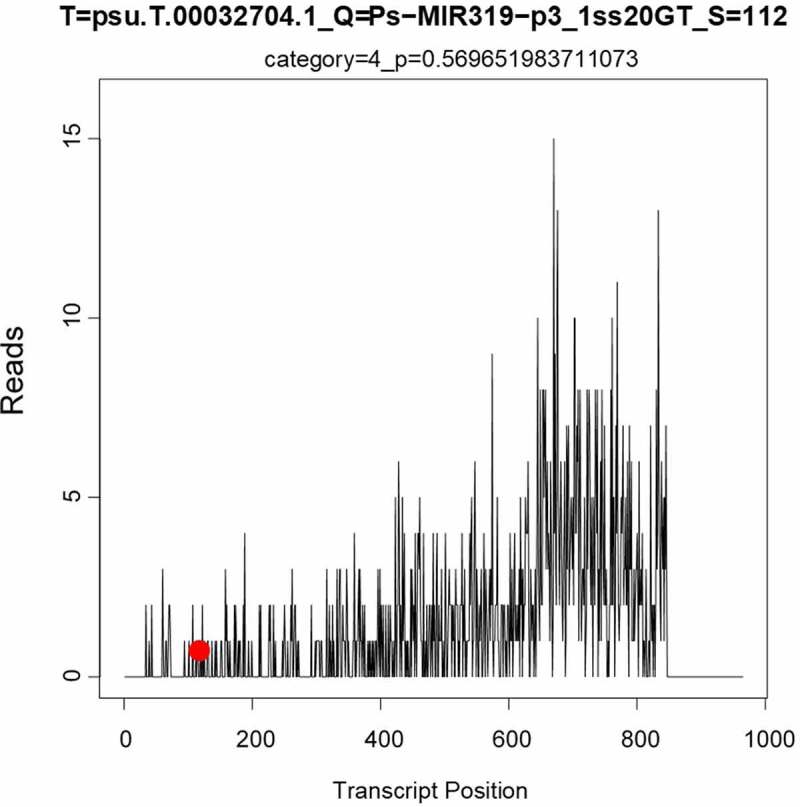
Target-plots of *PsmiR319*-regulated mRNA and degradome sequencing.

**Figure 6.**
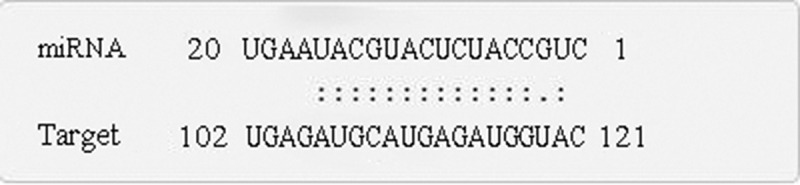
Specific nucleotide sequence of psu.T.00032704 that cleaved by *PsmiR319.*

The online software psRNATarget, which is suitable for prediction of target genes in plants, was used to predict the target genes of miR319 of several model plants (*A. thaliana, O. sativa*, and *N. tabacum*) and *Paeonia suffruticosa* (Table 2). The target genes of miR319 in *A. thaliana* were the transcription factors MYB and TCP, and aldehyde dehydrogenase (ALDH) synthesis gene. The target genes of miR319 in *O. sativa* included ARM repeat fold domain containing protein; transcription factors PCF6, PCF8, and MYB; 40S ribosomal protein; and proteins of Os03g0388900, Os08g0288000, Os11g0162300, Os07g0152000, and Os09g0483100. MiR319 target genes in *N. tabacum* were GAMyb-like1, Glycerol-3-phosphate ABC transporter permease protein, Pol protein, and Immuno-dominant variable surface antigen-like. The target gene in *Paeonia suffruticosa* was MYB_related transcription factor (psu.T.00032704), whose nucleotide sequence is 5’-ATTTATATTGATGTATAGTTTTTTCAATTTTTCAGGGGTCCTGGTGTAGCTGTGCTTGTCCTATCATGGGTGATTACTCTGTACACACTGTAGCAAATGGTTGAGATGCATGAGATGGTACCAGGGAAACGATTTGACAGATATCATGAGCTTGGTCAGCATGCTTTTGGAGAAAAGCTTGGCCTTTATATTGTGGTGCCCCAACAGCTTATTGTTGAAGTTGGTGTCAACATAGTCTATATGGTCACCGGAGGAAAATCCCTAAAGAAATTCCATGACACTGTCTGTAGCACCTGCAAACCAATCAAAACCACTTACTTCATTATGATCTTCTCCTCTGTTCATTTTGTGCTCTCCCACCTCCCCAACTTCAACTCCATTGCTGGTGTCTCTCTTGCTGCTGCAGTCATATCCTTAAGTTACTCAACTATTGCTTGGGGAGCAGCTATTGATAAGGGTGTTCAGCCAGATGTGCAATATGGCTACAAAGCCAAATCCACTTCAGGAACAATCTTCAACTTCTTTAGTGCCTTGGGAGAAGTTGCATTTGCTTATGCAGGGCACAATGTGGTGTTGGAGATTCAAGCAACAATTCCATCTACTCCAGAGAAGCCATCAAAGGGACCCATGTGGAGAGGAGTGATTGTTGCCTATATAGTAGTGGCTCTGTGTTACTTTCCAGTTGCTCTAATTGGGTATTGGATGTATGGAAATAGTGTTGAGGATAACATTCTAATTTCATTAAGTAAACCTGCTTGGCTGATTGCAATGGCCAACTTGTTTGTTGTCATCCATGTTATTGGAAGCTATCAGATCTATGCCATACCAGTGTTTGACATGATAGAAACTGTCTTGGTAAAGAAGCTGCATTTCACACCATATTTTACTCTTCTATATATAACAGGGAAAAAAAACCTTTTGACATTGTTGAGGACCCATTGAGTTGCGGCGGAAGCATCAGTTGC-3’. Target-plots (Figure 5) showed the mRNA cleavage sites within target genes silenced by *PsmiR319*. Figure 6 showed the specific nucleotide sequence of psu.T.00032704 that cleaved by *PsmiR319*, which indicates that *PsmiR319* plays a role in binding to the 102–121 nucleotide sequence of psu.T.00032704.

**Table 2.** Predicted target genes of miR319 and their functions.

Species	Acc of target gene	Function of target gene
*Arabidopsis thaliana*	AT2G26950.1	AtMYB104, MYB104 | myb domain protein 104
	AT5G06100.1	MYB33, ATMYB33 | myb domain protein 33
	AT5G06100.3	MYB33, ATMYB33 | myb domain protein 33
	AT3G11440.1	ATMYB65, MYB65 | myb domain protein 65
	AT5G06100.2	MYB33, ATMYB33 | myb domain protein 33
	AT2G31070.1	TCP10 | TCP domain protein 10
	AT3G66658.1	ALDH22A1 | aldehyde dehydrogenase 22A1
	AT3G66658.2	ALDH22A1 | aldehyde dehydrogenase 22A1
	AT3G15030.3	TCP4 | TCP family transcription factor 4
	AT3G15030.2	TCP4 | TCP family transcription factor 4
*Oryza sativa*	CT859118	Os03g0388900 protein
	TC543965	Os03g0388900 protein
	TC506068	Os08g0288000 protein
	TC483757	ARM repeat fold domain containing protein
	TC483756	Chromosome chr4 scaffold_39
	BE039775	40S ribosomal protein S24
	TC527879	Os11g0162300 protein
	TC539670	Transcription factor PCF6
	TC493949	Os07g0152000 protein
	TC508999	Transcription factor PCF8
	TC522883	Transcription factor PCF8
	TC490293	Chromosome chr9 scaffold_33
	CX108168	Os09g0483100 protein
	TC565219	Transcription factor GAMYB
*Nicotiana tabacum*	TC156902	Chromosome undetermined scaffold_247
	TC151380	GAMyb-like1
	AM811745	Glycerol-3-phosphate ABC transporter permease protein
	TC134794	Chromosome chr10 scaffold_43
	BP534314	Pol protein
	DW003240	Immuno-dominant variable surface antigen-like
*Paeonia suffruticosa*	psu.G.00032704	MYB_related

### 3.5 *Expression characteristics of* PsmiR319 *in different flower development stages of tree peony*

**Figure 7.**
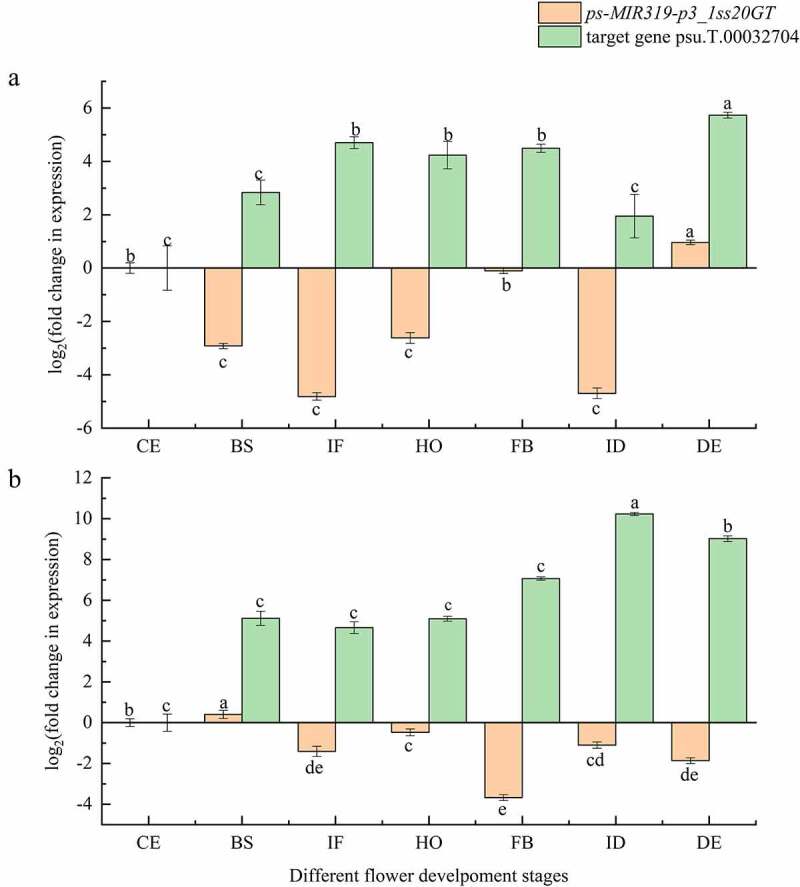
Relative expression of *PsmiR319* and its target gene among different flower development stages of tree peony.

The expression of both *PsmiR319* and its target gene were compared in tree peony varieties ‘Feng Dan’ and ‘Lian He’ using quantitative RT-PCR. The expression level of *PsmiR319* in ‘Feng Dan’ (Figure 7a) initially decreased and then increased from the period of CE to HO, with the highest expression found in IF phase. Expression of the target gene of *PsmiR319*-psu.T.00032704-showed the opposite trend, increasing initially and then decreasing at these periods, and the lowest expression was detected in IF phase. Therefore, there may be a negative expression regulation relationship between *PsmiR319* and psu.T.00032704. From the period of FB to DE, the expression content trend of *PsmiR319* was consistent with its target gene in ‘Feng Dan’. Expression of *PsmiR319* in the BS phase of ‘Lian He’ (Figure 7b) was the highest among all the phases, and expression trends of *PsmiR319* and its target genes in CE, BS, IF, HO, ID, and DE periods showed positive correlation and showed negative expression regulation in the FB phase in the plant. These results indicated that *PsmiR319* may play a role in the development of peony flowers by interacting with its target gene.

## 4 Discussion

In recent years, an increasing number of studies on how miRNA is involved in plant growth and development have been conducted to define the functions of miRNA in plants. The development of high-throughput sequencing technology has facilitated the discovery of increasing numbers of plant miR319s.^36^ At present, pre-miR319 of 41 plant species have been registered in the miRBase database, and these are distributed in four phyla, six classes, 20 orders, 22 families, and 40 genera, with plants such as *M. truncatula, V. unguiculata, A. thaliana*, and *M. domestica* containing multiple pre-miR319s (Table S1). miR319 is highly conserved, with representatives existing in various plants from moss to flowering plants, and different miR319s in the same plant may be derived from the same hairpin structure.^37^ However, the length of the registered pre-miR319 sequences examined in the current study were quite different (52–308 bp). The precursor sequence of *PsmiR319* of tree peony that was found in the present study is 60 nt, and therefore belongs to the shorter range of precursor sequences. Phylogenetic analysis revealed that pre-*PsmiR319* of tree peony and some of the precursor sequences of *L. usitatissimum* and *P. abies* clustered into one branch of the phylogenetic tree (Figure 2), with pre-*PsmiR319* of tree peony exhibiting the closest evolutionary relationship with that of *L. usitatissimum*, followed by that of *P. abies*, and this cluster is far from other angiosperms. It is speculated that the formation of pre-miR319 is affected by a variety of factors except for species differences. The same phenomenon was found during exploration of the evolutionary characteristics of miR395a in wild bananas.^38^

Comparative sequence analysis revealed that the number of mature miR319 sequences found in different plants varies, with the largest number of miRNAs in soybeans, reaching 17 (Table S2). The species *A. tauschii, A. lyrata, B. rapa, B. distachyon, M. truncatula, M. domestica, O. sativa, P. patens, S. tuberosum, S. Lycopersicum*, and *Z. mays* contain mature sequences from the 5p and 3p arms. The mature sequence of miR319 is highly conservative, but mature sequences from the 5p arm of pre-miR319 are markedly different (Figure 3). Conservation of miR319 has been described in multiple studies.^39,40^ *PsmiR319* of tree peony was able to form a typical hairpin structure that was derived from the 3p arm sequence and partial loop sequence of its precursor sequence (Figure 4).

Target gene prediction results (Table 2) of miR319 in *A. thaliana, O. sativa, N. tabacum*, and *P. suffruticosa* showed that TCP and MYB transcription factors were the predominant target gene of miR319. Degradation sequencing results (Figure 5) and sequence prediction of cleavage (Figure 6) in tree peony confirmed the target gene prediction results. *TCP* genes, first discovered in corn, snapdragon, and rice, encode a set of plant-specific transcription factors.^41^ TCP transcription factor regulated by miR319 is important regulators of plant growth and participates in the development and senescence of plant leaves, flowers, and male and female gametes.^41–44^ MYB transcription factors are one of the largest families of transcription factors in plants. In recent years, research has increasingly shown that MYB is instrumental in regulating the growth and development of plants, especially the process of flower development.^45,46,48^ As target genes of miR319, MYB family transcription factors influence the GA-dependent flowering pathway.^47^ *VcmiRNA319-VcMYBs* interact with abscisic acid in blueberry fruit maturation and participate in anthocyanin synthesis pathway.^49^ Expression of MYB transcription factor targeted by miR319 was reduced and lateral root formation was inhibited under boron toxicity, thereby limiting boron uptake and upward transport.^50^ In addition, MiR319 can target *MYB33* and *MYB65*.^51^ These findings suggest that the prediction of miR319 targeting MYB in this experiment is possible.

Flowering development is an extremely complex process that is regulated by multiple factors, including miR319.^52^ The fluorescence quantitative PCR analysis of *PsmiR319* expression levels in the current study (Figure 7a-b) showed an overall trend of initially increasing and then decreasing from the period CE to FB in the seven different flower development stages of ‘Feng Dan’ and presented a negative regulatory relationship with its target gene during these stages. In contrast, a positive correlation relationship between *PsmiR319* and its target gene occurred in the CE, BS, IF, HO, ID, and DE periods of ‘Lian He’. These observations indicate that *PsmiR319* and its corresponding target gene may interact to regulate flower development in tree peony. Mutants with over-accumulation of miR319 and downregulation of five class II TCP genes (namely *TCP2, TCP3, TCP4, TCP10*, and *TCP24*) exhibit a delayed flowering phenotype in *Arabidopsis*.^26^ TCP is regulated by miR319 and is a direct transcriptional activator of the photoperiod flowering regulator *CO*, which promotes the flowering of *A. thaliana* under the induced photoperiod.

## 5 Conclusion

Pre-miR319 was distributed in 41 plants, and the length of the precursor sequence was significantly different among plant species (52–308 bp). The sequence of pre-*PsmiR319* in tree peony was similar to those of spruce and flax, and formed a typical hairpin structure. The mature body is 20 bp long and located in the 3p arm and part of the loop sequence. The mature body sequence is highly conserved among different species. Expression of *PsmiR319* differed between early-flowering ‘Feng Dan’ and late-flowering ‘Lian He’ during flower developmental stages. *PsmiR319* and the target gene MYB-related transcription factor showed a negative expression regulation relationship during the periods of CE, BS, IF, and HO of early-flowering variety ‘Feng Dan’ and the same in FB (Full blooming) periods of late-flowering of ‘Lian He’. These findings lay a foundation for research on *PsmiR319* and provide resources for the breeding of new varieties of tree peony with selection of early- and late-flowering characteristics, as well as for under. The function of *PsmiR319* in tree peony needs to be verified by cloning and transgenic expression in model plants in future experiments. And these findings could open avenues for research in other woody plants as well, such as forest trees.

## Data Availability

The datasets generated during and/or analysed during the current study are listed in the text.

## References

[1] Zhang L, Guo DL, Guo LL, Guo Q, Wang HF, Hou XG. Construction of a high-density genetic map and QTLs mapping with GBS from the interspecific F1 population of *P. ostii* ‘Fengdan Bai’ and *P. suffruticosa* ‘Xin Riyuejin’. Sci Hortic. 2019;246:190–10. doi:10.1016/j.scienta.2018.10.039.

[2] Wang XJ, Liang HY, Guo DL, Guo LL, Duan XG, Jia QS, Hou XG. Integrated analysis of transcriptomic and proteomic data from tree peony (*P. ostii*) seeds reveals key developmental stages and candidate genes related to oil biosynthesis and fatty acid metabolism. Hortic. Res. 2019;6:111. doi:10.1038/s41438-019-0194-7. 31645965 PMC6804530

[3] Xue YQ, Liu R, Xue JQ, Wang SL, Zhang XX. Genetic diversity and relatedness analysis of nine wild species of tree peony based on simple sequence repeats markers. Hortic Plant J. 2021;7(6):579–588. doi:10.1016/j.hpj.2021.05.004.

[4] Zhou L, Wang Y, Peng Z. Molecular characterization and expression analysis of chalcone synthase gene during flower development in tree peony *(Paeonia suffruticosa)*. Afr J Biotechnol. 2011;10(8):1275–1284. doi:10.5897/AJB10.599.

[5] Xi HV, He YJ, Chen HY. Functional characterization of *SmbHLH13* in anthocyanin biosynthesis and flowering in eggplant. Hortic Plant J. 2021;7(1):73–80. doi:10.1016/j.hpj.2020.08.006.

[6] Zhang M, Yang QQ, Yuan X, Yan XL, Wang J, Cheng TR, Zhang QX. Integrating genome-wide association analysis with transcriptome sequencing to identify candidate genes related to blooming time in *Prunus mume*. Front. Plant Sci. 2021;12:690841. doi:10.3389/fpls.2021.690841. 34335659 PMC8319914

[7] Sheng Y, Hao ZD, Peng Y, Liu SQ, Hu LF, Shen YB, Shi JS, Chen JH. Morphological, phenological, and transcriptional analyses provide insight into the diverse flowering traits of a mutant of the relic woody plant *Liriodendron chinense*. Hortic. Res. 2021;8:174. doi:10.1038/s41438-021-00610-2. 34333549 PMC8325688

[8] Sriboon S, Li HT, Guo CC, Senkhamwong T, Dai C, Liu KD. Knock-out of *TERMINAL FLOWER 1* genes altered flowering time and plant architecture in *Brassica napus*. BMC Genet. 2020;21:1–13. doi:10.1186/s12863-020-00857-z. 32429836 PMC7236879

[9] Li XY, Bian HW, Song DF, Ma SY, Han N, Wang JH, Zhu MY. Flowering time control in ornamental gloxinia (*Sinningia speciosa*) by manipulation of miR159 expression. Ann Bot. 2013;111(5):791–799. doi:10.1093/aob/mct034. 23404992 PMC3631324

[10] Li XY, Guo F, Ma SY, Zhu MY, Pan WH, Bian HW. Regulation of flowering time via miR172-mediated *APETALA2-like* expression in ornamental gloxinia (*Sinningia speciosa*). JZUS-B. 2019;20(4):322–331. doi:10.1631/jzus.B1800003. PMC6454313 30932377

[11] Wang J, Long Y, Zhang JW, Xue MD, Huang GG, Huang K, Yuan QH, Pei XW. Combined analysis and miRNA expression profiles of the flowering related genes in common wild rice (*oryza rufipogon* Griff.). Genes Genomics. 2018;40(8):835–845. doi:10.1007/s13258-018-0688-y. 30047109 PMC6060991

[12] Feng L, Xia R, Liu YL. Comprehensive characterization of miRNA and *PHAS* loci in the diploid strawberry (*Fragaria vesca*) genome. Hortic Plant J. 2019;5(6):255–267. doi:10.1016/j.hpj.2019.11.004.

[13] Wei XC, Xu W, Yuan YX, Yao QJ, Zhao YY, Wang ZY, Jiang WS, Zhang XW. Genome-wide investigation of microRNAs and their targets in *Brassica rapa* ssp. *pekinensis* root with *Plasmodiophora brassicae* infection. Hortic Plant J. 2016;2(4):209–216. doi:10.1016/j.hpj.2016.11.004.

[14] Basso MF, Ferreira PCG, Kobayashi AK, Harmon FG, Nepomuceno AL, Molinari HBC, Grossi-de-Sa MF. MicroRNAs and new biotechnological tools for its modulation and improving stress tolerance in plants. Plant Biotechnol J. 2019;17(8):1482–1500. doi:10.1111/pbi.13116. 30947398 PMC6662102

[15] Pandita D, Wani SH. MicroRNA as a tool for mitigating abiotic stress in rice (*Oryza sativa* L.). Recent Approaches in Omics for Plant Resilience to Climate Change. Khudwani, SKUAST-Kashmir, India: Springer; 2019. 109–133. doi:10.1007/978-3-030-21687-0_6.

[16] Zhao ZL, Niu SY, Fan GQ, Deng MJ, Wang YL. Genome-wide analysis of gene and microRNA expression in diploid and autotetraploid *Paulownia fortunei* (Seem) Hemsl. under drought stress by transcriptome, microRNA, and degradome sequencing. Forests. 2018;9(2):88. doi:10.3390/f9020088.

[17] Chen XM. A microRNA as a translational repressor of *APETALA2* in *Arabidopsis* flower development. Sci. 2004;303(5666):2022–2025. doi:10.1126/science.1088060. PMC5127708 12893888

[18] Wang JW, Czech B, Weigel D. miR156-regulated SPL transcription factors define an endogenous flowering pathway in *Arabidopsis thaliana*. Cell. 2009;138(4):738–749. doi:10.1016/j.cell.2009.06.014. 19703399

[19] Wang ST, Sun XL, Hoshino Y, Yu Y, Jia B, Sun ZW, Sun MZ, Duan XB, Zhu YM. MicroRNA319 positively regulates cold tolerance by targeting *OsPCF6* and *OsTCP21* in rice (*Oryza sativa* L.). PloS one. 2014;9(3):e91357. doi:10.1371/journal.pone.0091357. 24667308 PMC3965387

[20] Jung JH, Seo YH, Seo PJ, Reyes JL, Yun J, Chua NH, Park CM. The *GIGANTEA*-regulated microRNA172 mediates photoperiodic flowering independent of *CONSTANS* in *Arabidopsis*. Plant Cell. 2007;19(9):2736–2748. doi:10.1105/tpc.107.054528. 17890372 PMC2048707

[21] Fahlgren N, Montgomery TA, Howell MD, Allen E, Dvorak SK, Alexander AL, Carrington JC. Regulation of *AUXIN RESPONSE FACTOR3* by *TAS3* ta-siRNA affects developmental timing and patterning in *Arabidopsis*. Curr Biol. 2006;16(9):939–944. doi:10.1016/j.cub.2006.03.065. 16682356

[22] Shi XP, Jiang FL, Wen JQ, Wu Z. Overexpression of *Solanum habrochaites* microRNA319d (sha-miR319d) confers chilling and heat stress tolerance in tomato (*S. lycopersicum*). BMC Plant Biol. 2019;19(1):214. doi:10.1186/s12870-019-1823-x. 31122194 PMC6533698

[23] Ma XL, Zhang XG, Zhao KK, Li FP, Li K, Ning LL, He JL, Xin ZY, Yin DM. Small RNA and degradome deep sequencing reveals the roles of microRNAs in seed expansion in peanut (*Arachis hypogaea* L.). Front Plant Sci. 2018;9:349. doi:10.3389/fpls.2018.00349. 29662498 PMC5890158

[24] Guo YL, Qin XT, Zhang B, Xu XJ, Li ZN, Li MY. Overexpression of miR319 in petunia (*Petunia* × *hybrida*) promotes *de novo* shoot organogenesis from leaf explants. Vitro Cell Dev Biol Plant. 2021;57(1):72–79. doi:10.1007/s11627-020-10063-2.

[25] Koyama T, Sato F, Ohme-Takagi M. Roles of miR319 and TCP transcription factors in leaf development. Plant Physiol. 2017;175(2):874–885. doi:10.1104/pp.17.00732. 28842549 PMC5619901

[26] Liu J, Cheng XL, Liu P, Li DY, Chen T, Gu XF, Sun JQ. MicroRNA319-regulated TCPs interact with FBHs and PFT1 to activate *CO* transcription and control flowering time in *Arabidopsis*. PLoS Genet. 2017;13(5):e1006833. doi:10.1371/journal.pgen.1006833. 28558040 PMC5469495

[27] Schommer C, Debernardi JM, Bresso EG, Rodriguez RE, Palatnik JF. Repression of cell proliferation by miR319-regulated TCP4. Mol Plant. 2014;7(10):1533–1544. doi:10.1093/mp/ssu084. 25053833

[28] Nag A, King S, Jack T. miR319a targeting of *TCP4* is critical for petal growth and development in *Arabidopsis*. Proc Natl Acad Sci USA. 2009;106(52):22534–22539. doi:10.1073/pnas.0908718106. 20007771 PMC2799693

[29] Rubio-Somoza I, Weigel D. Coordination of flower maturation by a regulatory circuit of three microRNAs. PLoS Genet. 2013;9(3):e1003374. doi:10.1371/journal.pgen.1003374. 23555288 PMC3610633

[30] Todesco M, Rubio-Somoza I, Paz-Ares J, Weigel D. A collection of target mimics for comprehensive analysis of microRNA function in *Arabidopsis thaliana*. PLoS Genet. 2010;6(7):e1001031. doi:10.1371/journal.pgen.1001031. 20661442 PMC2908682

[31] Khan M, Rozhon W, Poppenberger B. The role of hormones in the aging of plants-a mini-review. Gerontology. 2014;60(1):49–55. doi:10.1159/000354334. 24135638

[32] Zeng JB, Ye ZL, He XY, Zhang GP. Identification of microRNAs and their targets responding to low-potassium stress in two barley genotypes differing in low-K tolerance. J Plant Physiol. 2019;234:44–53. doi:10.1016/j.jplph.2019.01.011. 30665047

[33] Zhou M, Li DY, Li ZG, Hu Q, Yang CH, Zhu LH, Luo H. Constitutive expression of a miR319 gene alters plant development and enhances salt and drought tolerance in transgenic creeping bentgrass. Plant Physiol. 2013;161(3):1375–1391. doi:10.1104/pp.112.208702.0. 23292790 PMC3585603

[34] Zhou M, Luo H. Role of microRNA319 in creeping bentgrass salinity and drought stress response. Plant Signaling Behav. 2014;9(4):1375–1391. doi:10.4161/psb.28700. PMC4091478 24698809

[35] Zhang L, Song CW, Guo DL, Guo LL, Hou XG, Wang HF. Identification of differentially expressed miRNAs and their target genes in response to brassinolide treatment on flowering of tree peony (*Paeonia ostii*). Plant Signal. Behav. 2022;17(1):2056364. doi:10.1080/15592324.2022.2056364. 35343364 PMC8959526

[36] Si JN, Quan MY, Xiao L, Xie JB, Du QZ, Zhang DQ. Genetic interactions among Pto-miR319 family members and their targets influence growth and wood properties in *Populus tomentosa*. Mol Genet Genomics. 2020;295(4):855–870. doi:10.1007/s00438-020-01667-9. 32361785

[37] Sobkowiak L, Jarmolowski A, Karlowski W, Szweykowska-Kulinska Z. Non-canonical processing of *Arabidopsis* pri-miR319a/b/c generates additional microRNAs to target one RAP2. 12 mRNA isoform. Front Plant Sci. 2012;3:46. doi:10.3389/fpls.2012.00046. 22639648 PMC3355612

[38] Liu WH, Lin ZC, Liu YY, Li HS, Ni SS, Lin YL, Lai ZX. Cloning and evolution characteristics of pre-miR395a and promoter analysis in wild banana (*Musa itinerans*). Chin J Appl Environ Biol. 2018;24(1):0089–0096. doi:10.19675/j.cnki.1006-687x.2017.09042.

[39] Joshi G, Chauhan C, Das S. Microsynteny analysis to understand evolution and impact of polyploidization on MIR319 family within Brassicaceae. Dev Genes Evol. 2018;228(6):227–242. doi:10.1007/s00427-018-0620-0. 30242472

[40] Palatnik JF, Wollmann H, Schommer C, Schwab R, Boisbouvier J, Rodriguez R, Warthmann N, Allen E, Dezulian T, Huson D, et al. Sequence and expression differences underlie functional specialization of *Arabidopsis* microRNAs miR159 and miR319. Dev Cell. 2007;13(1):115–125. doi:10.1016/j.devcel.2007.04.012. 17609114

[41] Schommer C, Palatnik JF, Aggarwal P, Chételat A, Cubas P, Farmer EE, Nath U, Weigel D. Control of jasmonate biosynthesis and senescence by miR319 targets. PLoS Biol. 2008;6(9):e230. doi:10.1371/journal.pbio.0060230. 18816164 PMC2553836

[42] Balsemão-Pires E, Andrade LR, Sachetto-Martins G. Functional study of TCP23 in *Arabidopsis thaliana* during plant development. Plant Physiol Biochem. 2013;67:120–125. doi:10.1016/j.plaphy.2013.03.009. 23562796

[43] Fang YJ, Zheng YQ, Lu W, Li J, Duan YJ, Zhang S, Wang YP. Roles of miR319-regulated TCPs in plant development and response to abiotic stress. Crop J. 2021;9(1):17–28. doi:10.1016/j.cj.2020.07.007.

[44] Sarvepalli K, Nath U. Hyper-activation of the TCP4 transcription factor in *Arabidopsis thaliana* accelerates multiple aspects of plant maturation. Plant J. 2011;67(4):595–607. doi:10.1111/j.1365-313X.2011.04616.x. 21518050

[45] Liu S, Wang X, Li E, Douglas CJ, Chen JG, Wang S. R2R3 MYB transcription factor *PtrMYB192* regulates flowering time in *Arabidopsis* by activating *FLOWERING LOCUS C*. J Plant Biol. 2013;56(4):243–250. doi:10.1007/s12374-013-0135-1.

[46] Tominaga R, Iwata M, Sano R, Inoue K, Okada K, Wada T. *Arabidopsis CAPRICE-LIKE MYB 3* (*CPL3*) controls endoreduplication and flowering development in addition to trichome and root hair formation. Development. 2008;135(7):1335–1345. doi:10.1242/dev.017947. 18305006

[47] Wang BH , Sun, XX, Dong FY, Zhang F, Niu J-X. Cloning and expression analysis of an MYB gene associated with calyx persistence in korla fragrant pear. Plant Cell Rep. 2014;33(8):1333–1341. doi:10.1007/s00299-014-1619-2. 24756881

[48] Zhang LC, Liu GX, Jia JZ, Zhao GY, Xia C, Zhang LN, Li F, Zhang Q, Dong CH, Gao SC, et al. The wheat MYB-related transcription factor TaMYB72 promotes flowering in rice. J Integr Plant Biol. 2016;58(8):701–704. doi:10.1111/jipb.12461. 26714735

[49] Li XB, Hong Y, Jackson A, Guo FQ. Dynamic regulation of small RNAs in anthocyanin accumulation during blueberry fruit maturation. Sci. Rep. 2021;11(1):15080. doi:10.1038/s41598-021-93141-8. 34301985 PMC8302573

[50] Huang JH, Lin XJ, Zhang LY, Wang XD, Fan GC, Chen LS. Microrna sequencing revealed *citrus* adaptation to long-term boron toxicity through modulation of root development by mir319 and mir171. Int. J. Mol. Sci. 2019;20(6):1422. doi:10.3390/ijms20061422. PMC6470687 30901819

[51] Reichel M, Millar AA. Specificity of plant microRNA target *MIMICs*: cross-targeting of mir159 and mir319. J. Plant Physiol. 2015;180:45–48. doi:10.1016/j.jplph.2015.03.010. 25899728

[52] Zhu L, Li SS, Ma QY, Wen J, Yan KY, Li QZ. The *Acer palmatum* TCP transcription factor *ApTCP2* controls leaf morphogenesis, accelerates senescence, and affects flowering via miR319 in *Arabidopsis thaliana*. J Plant Growth Regul. 2021;41:244–256. doi:10.1007/s00344-021-10299-1.

